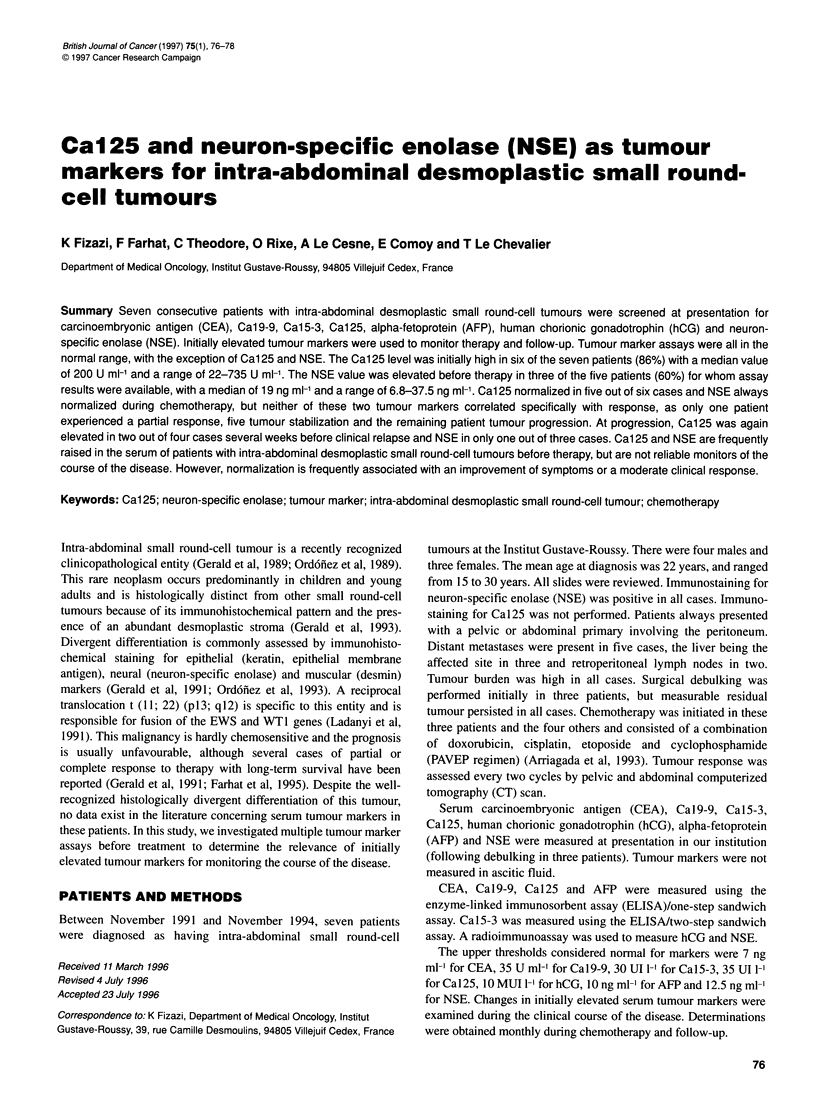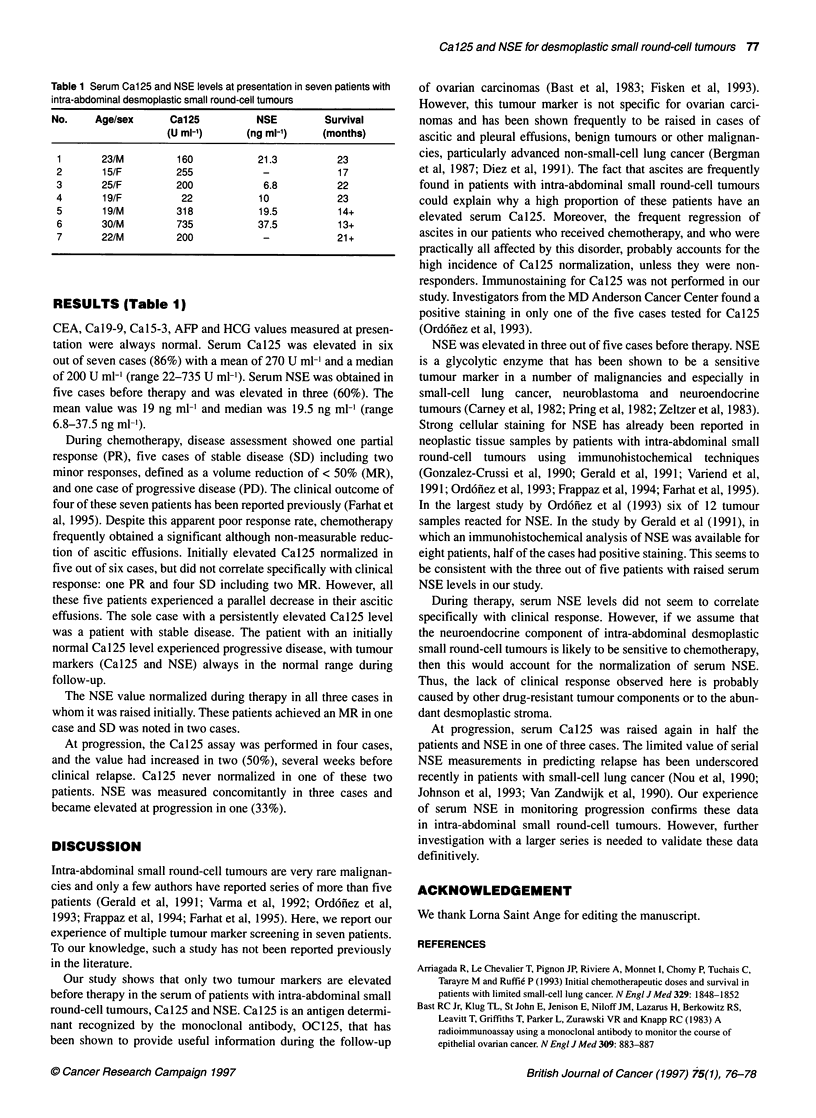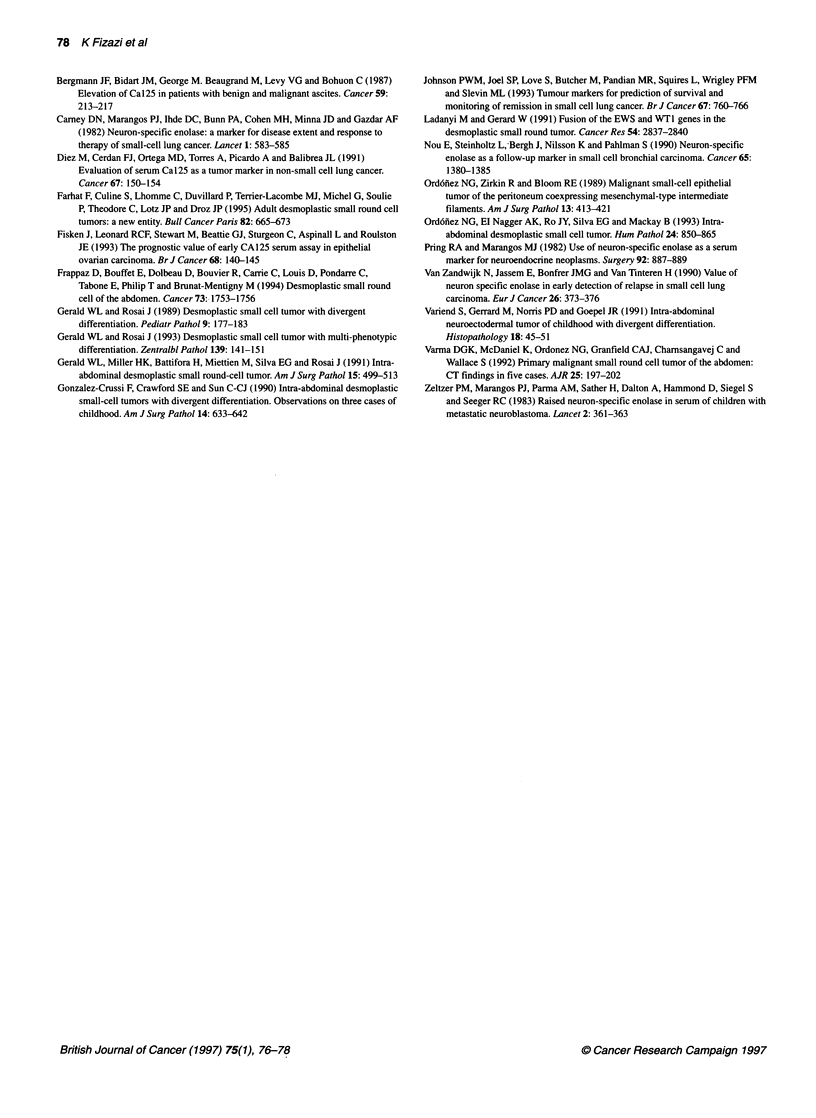# Ca125 and neuron-specific enolase (NSE) as tumour markers for intra-abdominal desmoplastic small round-cell tumours.

**DOI:** 10.1038/bjc.1997.12

**Published:** 1997

**Authors:** K. Fizazi, F. Farhat, C. Theodore, O. Rixe, A. Le Cesne, E. Comoy, T. Le Chevalier

**Affiliations:** Department of Medical Oncology, Institut Gustave-Roussy, Villejuif, France.

## Abstract

Seven consecutive patients with intra-abdominal desmoplastic small round-cell tumours were screened at presentation for carcinoembryonic antigen (CEA), Ca19-9, Ca15-3, Ca125, alpha-fetoprotein (AFP), human chorionic gonadotrophin (hCG) and neuron-specific enolase (NSE). Initially elevated tumour markers were used to monitor therapy and follow-up. Tumour marker assays were all in the normal range, with the exception of Ca125 and NSE. The Ca125 level was initially high in six of the seven patients (86%) with a median value of 200 U ml-1 and a range of 22-735 U ml-1. The NSE value was elevated before therapy in three of the five patients (60%) for whom assay results were available, with a median of 19 ng ml-1 and a range of 6.8-37.5 ng ml-1 . Ca1 25 normalized in five out of six cases and NSE always normalized during chemotherapy, but neither of these two tumour markers correlated specifically with response, as only one patient experienced a partial response, five tumour stabilization and the remaining patient tumour progression. At progression, Ca125 was again elevated in two out of four cases several weeks before clinical relapse and NSE in only one out of three cases. Ca125 and NSE are frequently raised in the serum of patients with intra-abdominal desmoplastic small round-cell tumours before therapy, but are not reliable monitors of the course of the disease. However, normalization is frequently associated with an improvement of symptoms or a moderate clinical response.


British Journal of Cancer (1997) 75(1), 76–78
© 1997 Cancer Research Campaign

### Ca125 and neuron-specific enolase (NSE) as tumour markers for intra-abdominal desmoplastic small round-cell tumours

**K Fizazi, F Farhat, C Theodore, O Rixe, A Le Cesne, E Comoy and T Le Chevalier**

Department of Medical Oncology, Institut Gustave-Roussy, 94805 Villejuif Cedex, France

**Summary** Seven consecutive patients with intra-abdominal desmoplastic small round-cell tumours were screened at presentation for carcinoembryonic antigen (CEA), Ca19-9, Ca15-3, Ca125, alpha-fetoprotein (AFP), human chorionic gonadotrophin (hCG) and neuron-specific enolase (NSE). Initially elevated tumour markers were used to monitor therapy and follow-up. Tumour marker assays were all in the normal range, with the exception of Ca125 and NSE. The Ca125 level was initially high in six of the seven patients (86%) with a median value of 200 U $ml^{-1}$ and a range of 22–735 U $ml^{-1}$. The NSE value was elevated before therapy in three of the five patients (60%) for whom assay results were available, with a median of 19 ng $ml^{-1}$ and a range of 6.8–37.5 ng $ml^{-1}$. Ca125 normalized in five out of six cases and NSE always normalized during chemotherapy, but neither of these two tumour markers correlated specifically with response, as only one patient experienced a partial response, five tumour stabilization and the remaining patient tumour progression. At progression, Ca125 was again elevated in two out of four cases several weeks before clinical relapse and NSE in only one out of three cases. Ca125 and NSE are frequently raised in the serum of patients with intra-abdominal desmoplastic small round-cell tumours before therapy, but are not reliable monitors of the course of the disease. However, normalization is frequently associated with an improvement of symptoms or a moderate clinical response.

**Keywords:** Ca125; neuron-specific enolase; tumour marker; intra-abdominal desmoplastic small round-cell tumour; chemotherapy

Intra-abdominal small round-cell tumour is a recently recognized clinicopathological entity (Gerald et al, 1989; Ordóñez et al, 1989). This rare neoplasm occurs predominantly in children and young adults and is histologically distinct from other small round-cell tumours because of its immunohistochemical pattern and the presence of an abundant desmoplastic stroma (Gerald et al, 1993). Divergent differentiation is commonly assessed by immunohistochemical staining for epithelial (keratin, epithelial membrane antigen), neural (neuron-specific enolase) and muscular (desmin) markers (Gerald et al, 1991; Ordóñez et al, 1993). A reciprocal translocation t (11; 22) (p13; q12) is specific to this entity and is responsible for fusion of the EWS and WT1 genes (Ladanyi et al, 1991). This malignancy is hardly chemosensitive and the prognosis is usually unfavourable, although several cases of partial or complete response to therapy with long-term survival have been reported (Gerald et al, 1991; Farhat et al, 1995). Despite the well-recognized histologically divergent differentiation of this tumour, no data exist in the literature concerning serum tumour markers in these patients. In this study, we investigated multiple tumour marker assays before treatment to determine the relevance of initially elevated tumour markers for monitoring the course of the disease.

### PATIENTS AND METHODS

Between November 1991 and November 1994, seven patients were diagnosed as having intra-abdominal small round-cell tumours at the Institut Gustave-Roussy. There were four males and three females. The mean age at diagnosis was 22 years, and ranged from 15 to 30 years. All slides were reviewed. Immunostaining for neuron-specific enolase (NSE) was positive in all cases. Immunostaining for Ca125 was not performed. Patients always presented with a pelvic or abdominal primary involving the peritoneum. Distant metastases were present in five cases, the liver being the affected site in three and retroperitoneal lymph nodes in two. Tumour burden was high in all cases. Surgical debulking was performed initially in three patients, but measurable residual tumour persisted in all cases. Chemotherapy was initiated in these three patients and the four others and consisted of a combination of doxorubicin, cisplatin, etoposide and cyclophosphamide (PAVEP regimen) (Arriagada et al, 1993). Tumour response was assessed every two cycles by pelvic and abdominal computerized tomography (CT) scan.

Serum carcinoembryonic antigen (CEA), Ca19-9, Ca15-3, Ca125, human chorionic gonadotrophin (hCG), alpha-fetoprotein (AFP) and NSE were measured at presentation in our institution (following debulking in three patients). Tumour markers were not measured in ascitic fluid.

CEA, Ca19-9, Ca125 and AFP were measured using the enzyme-linked immunosorbent assay (ELISA)/one-step sandwich assay. Ca15-3 was measured using the ELISA/two-step sandwich assay. A radioimmunoassay was used to measure hCG and NSE.

The upper thresholds considered normal for markers were 7 ng $ml^{-1}$ for CEA, 35 U $ml^{-1}$ for Ca19-9, 30 UI $l^{-1}$ for Ca15-3, 35 UI $l^{-1}$ for Ca125, 10 MUI $l^{-1}$ for hCG, 10 ng $ml^{-1}$ for AFP and 12.5 ng $ml^{-1}$ for NSE. Changes in initially elevated serum tumour markers were examined during the clinical course of the disease. Determinations were obtained monthly during chemotherapy and follow-up.

*Received 11 March 1996*
*Revised 4 July 1996*
*Accepted 23 July 1996*

*Correspondence to:* K Fizazi, Department of Medical Oncology, Institut Gustave-Roussy, 39, rue Camille Desmoulins, 94805 Villejuif Cedex, France

**Table 1** Serum Ca125 and NSE levels at presentation in seven patients with intra-abdominal desmoplastic small round-cell tumours

| No. | Age/sex | Ca125 (U ml$^{-1}$) | NSE (ng ml$^{-1}$) | Survival (months) |
|---|---|---|---|---|
| 1 | 23/M | 160 | 21.3 | 23 |
| 2 | 15/F | 255 | – | 17 |
| 3 | 25/F | 200 | 6.8 | 22 |
| 4 | 19/F | 22 | 10 | 23 |
| 5 | 19/M | 318 | 19.5 | 14+ |
| 6 | 30/M | 735 | 37.5 | 13+ |
| 7 | 22/M | 200 | – | 21+ |

### RESULTS (Table 1)

CEA, Ca19-9, Ca15-3, AFP and HCG values measured at presentation were always normal. Serum Ca125 was elevated in six out of seven cases (86%) with a mean of 270 U ml$^{-1}$ and a median of 200 U ml$^{-1}$ (range 22–735 U ml$^{-1}$). Serum NSE was obtained in five cases before therapy and was elevated in three (60%). The mean value was 19 ng ml$^{-1}$ and median was 19.5 ng ml$^{-1}$ (range 6.8–37.5 ng ml$^{-1}$).

During chemotherapy, disease assessment showed one partial response (PR), five cases of stable disease (SD) including two minor responses, defined as a volume reduction of < 50% (MR), and one case of progressive disease (PD). The clinical outcome of four of these seven patients has been reported previously (Farhat et al, 1995). Despite this apparent poor response rate, chemotherapy frequently obtained a significant although non-measurable reduction of ascitic effusions. Initially elevated Ca125 normalized in five out of six cases, but did not correlate specifically with clinical response: one PR and four SD including two MR. However, all these five patients experienced a parallel decrease in their ascitic effusions. The sole case with a persistently elevated Ca125 level was a patient with stable disease. The patient with an initially normal Ca125 level experienced progressive disease, with tumour markers (Ca125 and NSE) always in the normal range during follow-up.

The NSE value normalized during therapy in all three cases in whom it was raised initially. These patients achieved an MR in one case and SD was noted in two cases.

At progression, the Ca125 assay was performed in four cases, and the value had increased in two (50%), several weeks before clinical relapse. Ca125 never normalized in one of these two patients. NSE was measured concomitantly in three cases and became elevated at progression in one (33%).

### DISCUSSION

Intra-abdominal small round-cell tumours are very rare malignancies and only a few authors have reported series of more than five patients (Gerald et al, 1991; Varma et al, 1992; Ordóñez et al, 1993; Frappaz et al, 1994; Farhat et al, 1995). Here, we report our experience of multiple tumour marker screening in seven patients. To our knowledge, such a study has not been reported previously in the literature.

Our study shows that only two tumour markers are elevated before therapy in the serum of patients with intra-abdominal small round-cell tumours, Ca125 and NSE. Ca125 is an antigen determinant recognized by the monoclonal antibody, OC125, that has been shown to provide useful information during the follow-up of ovarian carcinomas (Bast et al, 1983; Fisken et al, 1993). However, this tumour marker is not specific for ovarian carcinomas and has been shown frequently to be raised in cases of ascitic and pleural effusions, benign tumours or other malignancies, particularly advanced non-small-cell lung cancer (Bergman et al, 1987; Diez et al, 1991). The fact that ascites are frequently found in patients with intra-abdominal small round-cell tumours could explain why a high proportion of these patients have an elevated serum Ca125. Moreover, the frequent regression of ascites in our patients who received chemotherapy, and who were practically all affected by this disorder, probably accounts for the high incidence of Ca125 normalization, unless they were non-responders. Immunostaining for Ca125 was not performed in our study. Investigators from the MD Anderson Cancer Center found a positive staining in only one of the five cases tested for Ca125 (Ordóñez et al, 1993).

NSE was elevated in three out of five cases before therapy. NSE is a glycolytic enzyme that has been shown to be a sensitive tumour marker in a number of malignancies and especially in small-cell lung cancer, neuroblastoma and neuroendocrine tumours (Carney et al, 1982; Pring et al, 1982; Zeltzer et al, 1983). Strong cellular staining for NSE has already been reported in neoplastic tissue samples by patients with intra-abdominal small round-cell tumours using immunohistochemical techniques (Gonzalez-Crussi et al, 1990; Gerald et al, 1991; Variend et al, 1991; Ordóñez et al, 1993; Frappaz et al, 1994; Farhat et al, 1995). In the largest study by Ordóñez et al (1993) six of 12 tumour samples reacted for NSE. In the study by Gerald et al (1991), in which an immunohistochemical analysis of NSE was available for eight patients, half of the cases had positive staining. This seems to be consistent with the three out of five patients with raised serum NSE levels in our study.

During therapy, serum NSE levels did not seem to correlate specifically with clinical response. However, if we assume that the neuroendocrine component of intra-abdominal desmoplastic small round-cell tumours is likely to be sensitive to chemotherapy, then this would account for the normalization of serum NSE. Thus, the lack of clinical response observed here is probably caused by other drug-resistant tumour components or to the abundant desmoplastic stroma.

At progression, serum Ca125 was raised again in half the patients and NSE in one of three cases. The limited value of serial NSE measurements in predicting relapse has been underscored recently in patients with small-cell lung cancer (Nou et al, 1990; Johnson et al, 1993; Van Zandwijk et al, 1990). Our experience of serum NSE in monitoring progression confirms these data in intra-abdominal small round-cell tumours. However, further investigation with a larger series is needed to validate these data definitively.

### ACKNOWLEDGEMENT

We thank Lorna Saint Ange for editing the manuscript.

### REFERENCES

Arriagada R, Le Chevalier T, Pignon JP, Riviere A, Monnet I, Chomy P, Tuchais C, Tarayre M and Ruffié P (1993) Initial chemotherapeutic doses and survival in patients with limited small-cell lung cancer. *N Engl J Med* **329**: 1848–1852

Bast RC Jr, Klug TL, St John E, Jenison E, Niloff JM, Lazarus H, Berkowitz RS, Leavitt T, Griffiths T, Parker L, Zurawski VR and Knapp RC (1983) A radioimmunoassay using a monoclonal antibody to monitor the course of epithelial ovarian cancer. *N Engl J Med* **309**: 883–887

© Cancer Research Campaign 1997 

Bergmann JF, Bidart JM, George M. Beaugrand M, Levy VG and Bohuon C (1987) Elevation of Ca125 in patients with benign and malignant ascites. *Cancer* **59**: 213–217

Carney DN, Marangos PJ, Ihde DC, Bunn PA, Cohen MH, Minna JD and Gazdar AF (1982) Neuron-specific enolase: a marker for disease extent and response to therapy of small-cell lung cancer. *Lancet* **1**: 583–585

Diez M, Cerdan FJ, Ortega MD, Torres A, Picardo A and Balibrea JL (1991) Evaluation of serum Ca125 as a tumor marker in non-small cell lung cancer. *Cancer* **67**: 150–154

Farhat F, Culine S, Lhomme C, Duvillard P, Terrier-Lacombe MJ, Michel G, Soulie P, Theodore C, Lotz JP and Droz JP (1995) Adult desmoplastic small round cell tumors: a new entity. *Bull Cancer Paris* **82**: 665–673

Fisken J, Leonard RCF, Stewart M, Beattie GJ, Sturgeon C, Aspinall L and Roulston JE (1993) The prognostic value of early CA125 serum assay in epithelial ovarian carcinoma. *Br J Cancer* **68**: 140–145

Frappaz D, Bouffet E, Dolbeau D, Bouvier R, Carrie C, Louis D, Pondarre C, Tabone E, Philip T and Brunat-Mentigny M (1994) Desmoplastic small round cell of the abdomen. *Cancer* **73**: 1753–1756

Gerald WL and Rosai J (1989) Desmoplastic small cell tumor with divergent differentiation. *Pediatr Pathol* **9**: 177–183

Gerald WL and Rosai J (1993) Desmoplastic small cell tumor with multi-phenotypic differentiation. *Zentralbl Pathol* **139**: 141–151

Gerald WL, Miller HK, Battifora H, Miettien M, Silva EG and Rosai J (1991) Intra-abdominal desmoplastic small round-cell tumor. *Am J Surg Pathol* **15**: 499–513

Gonzalez-Crussi F, Crawford SE and Sun C-CJ (1990) Intra-abdominal desmoplastic small-cell tumors with divergent differentiation. Observations on three cases of childhood. *Am J Surg Pathol* **14**: 633–642

Johnson PWM, Joel SP, Love S, Butcher M, Pandian MR, Squires L, Wrigley PFM and Slevin ML (1993) Tumour markers for prediction of survival and monitoring of remission in small cell lung cancer. *Br J Cancer* **67**: 760–766

Ladanyi M and Gerard W (1991) Fusion of the EWS and WT1 genes in the desmoplastic small round tumor. *Cancer Res* **54**: 2837–2840

Nou E, Steinholtz L, Bergh J, Nilsson K and Pahlman S (1990) Neuron-specific enolase as a follow-up marker in small cell bronchial carcinoma. *Cancer* **65**: 1380–1385

Ordóñez NG, Zirkin R and Bloom RE (1989) Malignant small-cell epithelial tumor of the peritoneum coexpressing mesenchymal-type intermediate filaments. *Am J Surg Pathol* **13**: 413–421

Ordóñez NG, El Nagger AK, Ro JY, Silva EG and Mackay B (1993) Intra-abdominal desmoplastic small cell tumor. *Hum Pathol* **24**: 850–865

Pring RA and Marangos MJ (1982) Use of neuron-specific enolase as a serum marker for neuroendocrine neoplasms. *Surgery* **92**: 887–889

Van Zandwijk N, Jassem E, Bonfrer JMG and Van Tinteren H (1990) Value of neuron specific enolase in early detection of relapse in small cell lung carcinoma. *Eur J Cancer* **26**: 373–376

Variend S, Gerrard M, Norris PD and Goepel JR (1991) Intra-abdominal neuroectodermal tumor of childhood with divergent differentiation. *Histopathology* **18**: 45–51

Varma DGK, McDaniel K, Ordonez NG, Granfield CAJ, Charnsangavej C and Wallace S (1992) Primary malignant small round cell tumor of the abdomen: CT findings in five cases. *AJR* **25**: 197–202

Zeltzer PM, Marangos PJ, Parma AM, Sather H, Dalton A, Hammond D, Siegel S and Seeger RC (1983) Raised neuron-specific enolase in serum of children with metastatic neuroblastoma. *Lancet* **2**: 361–363

 © Cancer Research Campaign 1997